# Insects as food and medicine: a sustainable solution for global health and environmental challenges

**DOI:** 10.3389/fnut.2023.1113219

**Published:** 2023-06-14

**Authors:** Owusu Fordjour Aidoo, Jonathan Osei-Owusu, Kwasi Asante, Aboagye Kwarteng Dofuor, Belinda Obenewa Boateng, Shadrack Kwaku Debrah, Kodwo Dadzie Ninsin, Shahida Anusha Siddiqui, Shaphan Yong Chia

**Affiliations:** ^1^Department of Biological Sciences, University of Environment and Sustainable Development, Somanya, Ghana; ^2^Department of Physical and Mathematical Sciences, University of Environment and Sustainable Development, Somanya, Ghana; ^3^Coconut Research Programme, Council for Scientific and Industrial Research, Sekondi, Ghana; ^4^Department of Horticulture and Crop Production Sunyani, University of Energy and Natural Resources, Sunyani, Ghana; ^5^Technical University of Munich, Campus Straubing for Biotechnology and Sustainability, Straubing, Germany; ^6^German Institute of Food Technologies (DIL e.V.), Quakenbrück, Germany; ^7^Laboratory of Entomology, Wageningen University & Research, Wageningen, Netherlands

**Keywords:** edible insects, entomophagy, entomotherapy, food security, insect-based foods, ecosystem function

## Abstract

Insects are a significant source of food for millions of people worldwide. Since ancient times, insects in medicine have been contributing to the treatment of diseases in humans and animals. Compared to conventional animal farming, the production of insects for food and feed generates significantly less greenhouse gas emissions and uses considerably less land. Edible insects provide many ecosystem services, including pollination, environmental health monitoring, and the decomposition of organic waste materials. Some wild edible insects are pests of cash crops. Thus, harvesting and consuming edible insect pests as food and utilizing them for therapeutic purposes could be a significant progress in the biological control of insect pests. Our review discusses the contribution of edible insects to food and nutritional security. It highlights therapeutic uses of insects and recommends ways to ensure a sustainable insect diet. We stress that the design and implementation of guidelines for producing, harvesting, processing, and consuming edible insects must be prioritized to ensure safe and sustainable use.

## 1. Introduction

The global population is rising exponentially, and so is the societal difficulty of meeting nutritional needs, which drives up the worldwide demand for meat (1). As a result, dietary diversity, biofortification, supplementation, and commercial food fortification, are all approaches that are beneficial in combating malnutrition (2–5). Thousands of insect species are consumed annually, mostly in developing nations (6). About 2.5 billion people worldwide rely on insects as a supplementary food source (7). Over the past decade, edible insects have gained popularity as healthy and environmentally friendly substitutes for traditional meat and dairy products. The global edible insect industry will be worth over $3 billion by 2030 (8). However, the consumption of insects is still unusual in most western populations if not considered a taboo (9–11). Many nations in Asia, Africa, Europe, and Latin America consume whole, easily recognizable edible insects as a typical snack or even as their primary source of protein (12). These insects are often prepared by being boiled, dried, toasted, or fried before being used in cooking (13).

Edible insects can solve many environmental and health issues, including climate change, hunger, and environmental degradation caused by agro-industrial production (14). The growing population of the world, along with the resulting shifts in demographics regarding lifestyle, dietary preference, and income, and the resulting expectation of increased access to a wide variety of lifestyle options has led to a heightened awareness of the global sustainability challenges humanity faces today (15). Sustainable development and commercialization require multi-disciplinary research into edible species and documentation of the species’ preparation process and therapeutic characteristics (16).

Though consuming edible insects for food and using them for treating animal and human diseases have received greater attention, there needs to be more information on other benefits associated with production, marketing and harvesting. For instance, the African coconut beetle *Oryctes monoceros* (Olivier), Asiatic rhinoceros beetles *Oryctes rhinoceros* (Linnaeus), and African palm weevil *Rhychophorus phoenicis* (L.) attack and kill economically important crops, such as palms, banana, and pineapple (17–20). However, many people in Sub-Saharan Africa consume the same insects because of their nutritional properties (21, 22).

This review discusses wild edible insects as agricultural pests of cash crops and how harvesting these insect pests could contribute to their management. Moreover, our review examines edible insects as a long-term solution to global food security by considering their nutritional properties, ecosystem services, and environmental impacts. We highlight the potential of wild edible insects as reservoirs for pathogens harmful to plants, animals, and humans. Furthermore, we discuss some edible insects with therapeutic properties for treating diseases. We highlight options for designing and implementing guidelines for using insects as food and the need to prioritize harvesting and consumption to ensure safe and sustainable use.

## 2. Methodology

**Figure 1:**
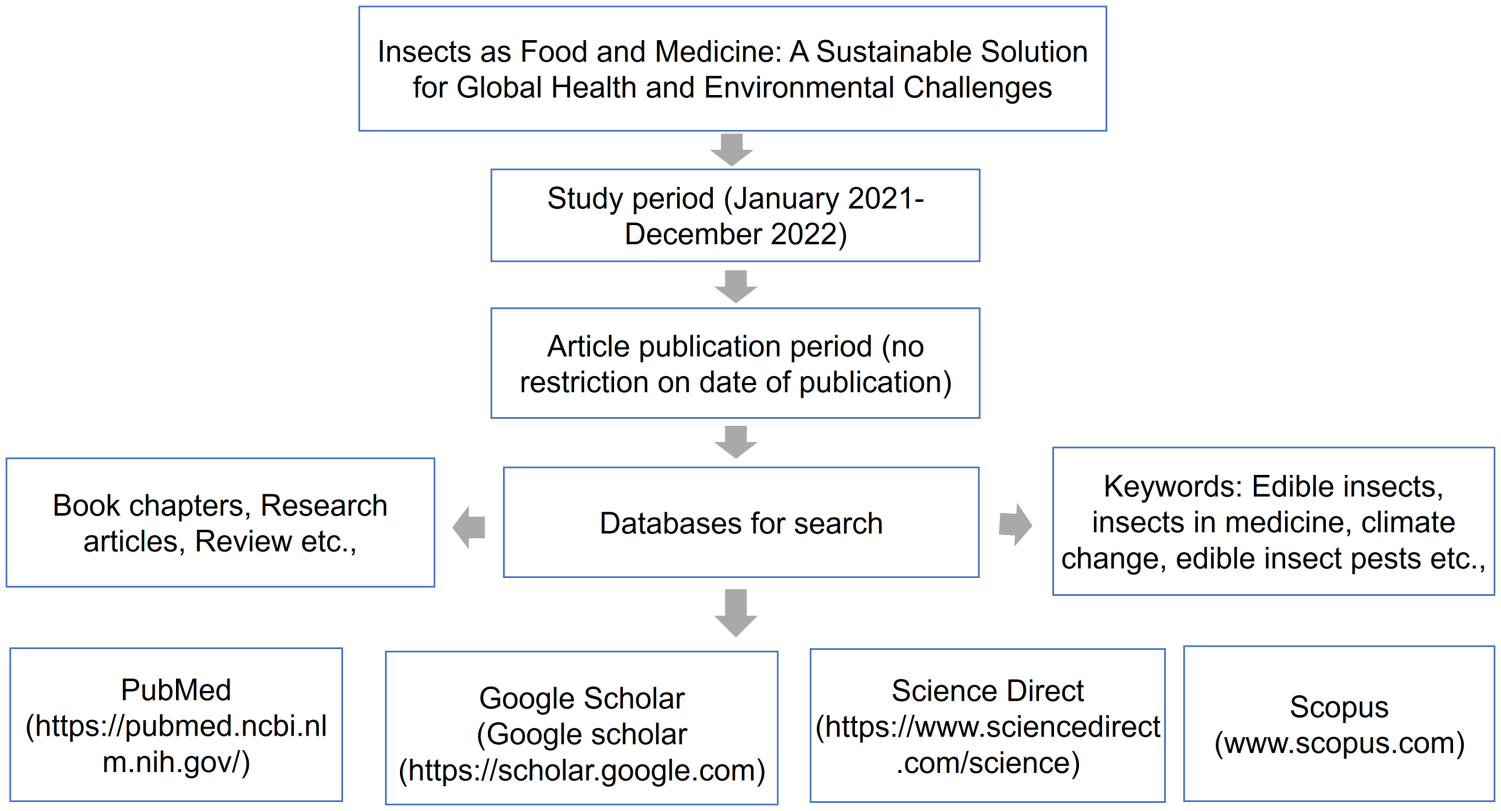
A schematic diagram showing the methodology followed during the review.

In this review, we sourced articles from these databases: Semantic Scholar^1^, Google Scholar^2^, Scopus^3^, Science Direct^4^, and SciELO^5^. The review started from January 2022 to December 2022. Articles published in indexed scientific journals and books were considered without limitations on the year of publication. We selected only articles published in English. To be more specific in our search, the keywords used, included “edible insects,” “the bioactivity of edible insects,” “edible insects and climate change,” “pathogens of edible insects,” “insects in medicine,” “nutritional benefits of insects,” “insects as food and feed,” “ecological benefits of edible insects,” “edible insect pests,” and “edible insects as vectors of diseases in plants, animals and crops.” A detailed representation of the search of articles for the review is illustrated in Figure 1.

## 3. Food and nutritional benefits of edible insects

Food security measures the availability and accessibility to safe, nutritious, and sufficient food. One billion people rely on livestock for their livelihood, and 70% of the 880 million rural poor who earn less than USD $1.00 a day rely on livestock at least in part for their income and food security (23). Nevertheless, the prevalence of undernourishment has increased by 1.5% points in recent years, representing a midpoint of about 720 to 811 million people suffering from hunger in the first year of the COVID-19 pandemic (24). The rise was estimated to be 446 million in Africa, 57 million in Asia, and 14 million in Latin America and the Caribbean (23–25). Substantial dietary changes are required to achieve global food security goals. Edible insects could serve as an alternative source of nutrients and are currently considered as significant food sources.

About 5.5 million insect species are available worldwide, of which approximately 1 million have been described (26). Of this number, approximately 2,100 species are edible (27). Among these edible insects are beetles, caterpillars, ants, bees, wasps, grasshoppers, true bugs, dragonflies, termites, and cockroaches (27). In Africa, for instance, the most critical edible insect orders include Lepidoptera (30.93%), Orthoptera (22.80%), Coleoptera (19.70%), Heteroptera (9.32%), Blattodea (7.40%), Hymenoptera (6.78%), Diptera (1.06%), Dictyoptera (0.85%), Odonata (0.64%), and Ephemenoptera (0.42%), with Odonata and Ephemeroptera forming relatively lower percentages (28).

Generally, it is difficult to determine the nutritional profile of edible insects due to the considerable differences encountered across species, country, insect feed composition, insect rearing mode, and developmental stage of insects, all of which may affect the nutritional profile. However, proteins, lipids, chitin, minerals, and vitamins form significant components of nutrients in edible insects. Protein represents the major component of nutrient composition in edible insects, followed by lipids (29). In terms of dry matter, the protein content of edible insects ranges between 35.3 and 61.3% for Blattodea (termites) and Orthoptera (crickets, grasshoppers, locusts), respectively (30). The latter exhibits the lowest lipid content of about 13.41% dry matter, whereas beetles, termites, and fly larvae depict high contents of approximately 33.40% dry matter. The former includes palmitic, stearic, palmitoleic, oleic, linoleic, α-linolenic and γ-linolenic acids. Chitin, the main polysaccharide component of the insect exoskeleton, protects insects from harsh environmental conditions. Furthermore, even though edible insects are generally rich in copper, iron, magnesium, manganese, phosphorous, selenium, and zinc, little is known about the nutritional profile of vitamins and minerals (31). The potential contribution of insects to food and nutritional security is crucial worldwide. However, much more knowledge is essential for the quantitative nutritional assessment of insects, especially vitamins and minerals. The nutritional profile of insects used as animal feed also needs more research.

## 4. Medicinal benefits of edible insects

In many cultures worldwide, traditional medicine has used insects for a long time to treat stomach aches, respiratory issues, and wound healing. However, most of the research on the functional significance of edible insects has been on their ability to act as antioxidants in cell models or *in vitro* (32). Additionally, there is scant research on how edible insects affect platelet aggregation, anti-inflammation, lipid modulation, and glucose metabolism (33–35). However, there has been a recent uptick in research on the potential health benefits of edible insects from a theoretical and practical standpoint (32). Recent advances have investigated the biological activities of common insect orders, such as Blattodea, Coleoptera, Diptera, and Hemiptera (Table 1).

**Table 1:** Common edible insects, isolated compounds and biological activity.

Insect	Common name	Compound isolated from	Isolated compound	Biological activity	References
Blattodea
*Periplaneta americana* (Linnaeus)	American cockroach	Adult	Isocoumarins periplatins A–D	Cytotoxic activities against human liver (HepG2) and breast cancer (MCF-7) cells	(36)
*Polyphaga plancyi* (Bolivar)	Chinese cockroach	Not determined	Plancyamide A Plancypyrazine A Plancypyrazine B Plancyol A	Antiproliferative	(37)
*Macrotermes* spp.	Termites	Body surface	Roseoflavin, 8-methylamino-8- demethyl-D-riboflavin Natalamycin Termisoflavones A-C	Antibacterial Antifungal	(38, 39)
Coleoptera
*Blaps japanensis*		Adult	Blapsols A–D	Anti-inflammatory	(40)
*Holotrichia diomphalia* (Hope)		Larvae	Tricin, palmitinic acid eicosane	Antifungal	(41)
*Hycleus oculatus* (Thunberg) *H. tinctus* (Walsingham) *H. lunata* (Smith)	African blister beetles	Adult	(*R*)- (+)-palasonin Palasonimide Cantharimide Palasonin	Anticancer	(42, 43)
Diptera
*Hermetia Illucens* (L.)	Black Soldier Fly	Larvae	α-pyrone diketopiperazine	Antibacterial	(44)
Hemiptera					
*Aspongopus* *Chinensis* (Dallas)	Chinese stinkbug	Not determined	(±)-Aspongamide A	Therapeutic agents for chronic kidney disease	(45, 46)
*Dactylopius* *coccus* (Costa)		Not determined	Carminic acid	Antioxidant	(40)
Hymenoptera
*Perga affinis* (Kirby)	Australian sawfly	Larvae	Macrocarpal and grandinol	Antimicrobial against *Bacillus subtilis*	(47)
*Polyrhachis dives* (Smith)	Chinese black ants	Adult	(±)-Polyrhadopamine A, (±)-Polyrhadopamine B, (±)-Polyrhadopamine C, trolline, (±)-Polyrhadopamine C *β*-carboline-3-carboxamide 5-(3-indolylmethyl)-nicotinsaureamide	Used to treat rheumatoid and osteo arthritis, inflammatory diseases, and central nervous system. Antiproliferative against T-lymphocytes	(48, 49)
*Tetraponera rufonigra* (Jerdon)	Iron ant	Adult	Tetraponerines	Neurotoxic Antiproliferative	(50)
*Apis mellifera* (Linnaeus)	Brazilian red propolis	Not determined	Lupeol, lupenone, lupeol acetate	Antitumor	(51)
Lepidoptera
*Byasa polyeuctes* (Doubleday)	Taiwan butterfly	Adult	Papilistatin	Antibacterial Anticancer	(52)
Orthoptera
*Brachystola magna* (Girard)	Texas grasshopper	Adult	Pancratistatin Narciclasine Ungeremine	Anticancer	(40)
*Schistocerca gregaria* (Forsskål)	Desert locust	Adult gut	Desmosterol, (3β, 5α) cholesta-8, 14, 24-trien-3-ol, 4, 4-dimethyl, (3β, 20R) cholesta-5, 24-dien-3, 20-diol	Antimicrobial properties under investigation	(53)

Numerous therapeutic qualities, such as antioxidant and anti-platelet aggregation action, have been examined *in vitro* in various edible insects (32). The expression of anti-tryptic and chymotryptic activity, as well as the inhibition of pancreatic lipase, and dipeptidyl peptidase-4 activity, have all been studied *in vitro* (32). Moreover, a recent study showed that flour from *Tenebrio molitor* (Linnaeus) affected the growth of Lactobacillus and Bifidobacterium by improving short-chain fatty acid production and viability in nutritive stress conditions (54).

There have been parallel efforts in cellular and *ex vivo* models. Human umbellar vein endothelial cells (HUVECs) exhibited lower levels of thrombin, plasminogen activator inhibitor, and factor Xa after exposure to indole alkaloids derived from *Protaetia brevitaris seulensis* (Kolbe) (55). The ethanolic extract from *Gryllus bimaculatus* (De Geer), *Oxya chinensis sinuosa* (Mishchenko), and *Protaetia brevitaris seulensis* reduced intracellular lipid accumulation and triacylglycerol in liver hepatocellular carcinoma (HepG2) (55). Lipid accumulation was also reduced in L-02 cells, a human fetal hepatocyte line, by polyunsaturated fatty acids and α-linolenic acid from *Bombyx mori* (L.) (35). Again, tetrahydroquinolines isolated from *Allomyrna dichotoma* caused a reduction in vascular cell adhesion molecule-1 and intercellular adhesion molecule-1 levels, as well as adherence of monocytes to HUVECs monolayers and migration of human neutrophils (56).

Moreover, there have been investigations into the effects of edible insects through animal models. For instance, Zebrafish *Danio rerio* (F. Hamilton) fed with *Hermetia illucens* (L.) showed a significant increase in growth rate (57). Tetrahydroquinolines from *Allomyrina dichotoma* (L.) administered to septic C57BL/6 mice increased their survival rate (56). Also, wheat noodles enriched with *B. mori* powder significantly reduced post-prandial blood glucose, glucose peak, and area under the curve of glucose and glucose index (58).

## 5. Contribution of edible insects to climate change mitigation

Rapid global climate change continues to threaten the existence of humanity on earth (59). Nevertheless, the production of livestock is solely responsible for more than 14.5% of all greenhouse gas (GHG) emissions (CO_2_, CH_4_, and NO_2_), 64% of the world’s NH_4_ emissions, water pollution, and biodiversity loss (7). Livestock, a significant driver of environmental degradation, calls for an alternative protein source, such as consuming edible insects known for their low contribution to GHG (60). For example, the emissions of greenhouse gases per kilogram of mass and NH_3_ of three edible insects *T. molitor*, *A. domesticus*, and *Locusta migratoria* (L.) were lower than pigs and far lower than cattle (61). Also, the global warming potential per kg of *Protaetia brevitarsis seulensis* production (15.93 kgCO_2_eq) was lower than the conventional meat sources, such as chicken (18–36 kgCO_2_eq), pork (21–53 kgCO_2_eq), and beef (75–170 kgCO_2_eq) (62). Furthermore, the methane output of cockroaches and beetle larvae was more than 20 times lower than that of cattle and was similar to or slightly lower than that of pigs (62). Using the lifecycle assessment (LCA) method, GHG emission from producing 1 kg of mealworm was far lower than that of chicken, pork, or beef (63). Also, the LCA for black soldier fly production was more sustainable than that of fresh chicken meat (64).

## 6. Contribution of edible insects to ecosystem services

Edible insects provide direct and indirect ecosystem services like cultural, provision, maintenance, and regulation as per the definition by the Millennium Ecosystem Services (65). One of the ecological services that insects may give is the decomposition of organic waste. Insects are frequently employed to break down agricultural and culinary waste when raising insects in large numbers for food. The black soldier fly, Mealworms, houseflies, and crickets are the most effective bio converters (7, 66, 67). These insects can simultaneously make valuable commodities, like biomass from insects, cosmetics, lubricants, medicines, biofuels, surfactants and fertilizers (68). These insects also render regulatory services through the control of crop pests and pollination (Table 2).

**Table 2:** Examples of insects’ species and the ecosystem services they render.

Order	Family	Species	Pollination	Decomposition	Reduction of food waste	Food chain or food web	Reference
Blatodea	Termitidae	*Macrotermes* spp.		√		√	(69)
	Blattidae	*Periplaneta americana* (L.)		√		√	
Coleoptera	Curculionidae	*Rhynchophorus ferrugineus* (Olivier)	√	√		√	(70–72)
	Dytiscidae	*Schoolhouse acupunctatus* (Gyllenhal)	√	√		√	
		*Dytiscus latissimus* (L.)	√	√		√	
	Hydrophilidae	*Cybister* spp.	√	√		√	
	Curculionidae	*Metamasius* spp.	√	√		√	
	Scarabaeidae	*Anoplognathus viridiaeneus* (Donovan)	√	√		√	
		*Phyllophaga* spp.	√	√		√	
		*Oryctes* spp.	√			√	
	Tenebrionidae	*Tenebrio* spp.	√	√		√	
		*Zophobas morio* (Fabr.)	√	√		√	
	Lucanidae	*Lucanus cervus* (L.)	√	√		√	
Lepidoptera	Cossidae	*Comadia* spp.	√	√	√	√	(73, 74)
		*Redtenbacheri* spp.	√	√	√	√	
	Hesperiidae	*Aegiale hesperiaris* (Walker)	√	√	√	√	
	Saturniidae	*Cirina butyrospermi* (Vuillet)	√	√	√	√	
		*Gonimbrasia belina* (Westwood)	√	√	√	√	
	Psychidae	*Psychidae* gen.	√	√	√	√	
	Crambidae	*Omphisa fuscidentalis* (Hampson)	√	√	√	√	
	Sphingidae	*Macroglossum stellatarum* (L.)	√	√	√	√	
	Bombycidae	*Bombyx mori* (L.)	√	√	√	√	
Hymenoptera	Vespidae	*Vespula vulgaris* (L.)	√				(75)
	Formicidae	*Oecophylla smaragdina* (Fabr.)	√	√		√	(76, 77)
		*Camponotus inflatus* (Lubbock)	√	√		√	
		*Oecophylla longinoda*	√	√		√	
		(Latreille)	√	√		√	
		*Myrmelachista schumanni* (Emery)	√	√		√	
		*Atta* spp.	√			√	
	Apidae	*Apis* spp.	√			√	
		*Bombus* spp.	√			√	
		*Xylocopa* spp.	√			√	
		*Trigona* spp.	√			√	
Orthoptera	Pyrgomorphidae	*Sphenarium purpurascens* (Charpentier)				√	(78–81)
	Acrididae	*Locusta migratoria* (L.)				√	
	Gryllidae	*Oxya hyla* (Serville)				√	
		*Acheta domestica* (L.)			√	√	
	Gryllidae	*Gryllus veletis* (Alexander & Bigelow)			√	√	
	Tettigonioidea	*Tettigonia viridissima* (L.)			√	√	
Hemiptera	Aphididae	*Aphididae* spp.				√	(71)
	Cicadidae	*Magicicada* spp.				√	

*√ indicates the service rendered.

### 6.1. Pollination

Globally, insects are relied on extensively for pollination in agriculture. This ecosystem service is of great economic value. The Apidae family of bees is considered the most significant edible insect pollinators. Honey bees are also noted to increase the yield of about 96% of crops, with recent literature indicating that wild bees might be even better pollinators than honey bees (82). The study further indicated that, in the United States, bee pollination services are valued at 3.07 billion US dollars. Therefore, the limitation on pollination by bees poses a significant risk to yield stability and food security (82). Butterflies and moths are also important pollinators of crops. *Agrius convolvuli* (L.), a Hawk moth, is a critical papaya pollinator in Kenya and Southeast Asia (64).

## 7. Decomposition

Edible insects like ants and termites are vital in soil formation and the cycling of nutrients through the decomposition of organic matter. The direct consumption of organic matter and the indirect effects of insect activities, such as creating larval tunnels in woody materials, result in their decomposition in the tropical forest, thereby increasing soil fertility. An example is the palm weevil which deposits its eggs on the trunk or directly on the inner tissues of falling trees. The emergent larvae then accelerate the decomposition of the logs by burrowing through and feeding on the inner tissues. Twenty-nine percent of deadwood’s carbon flux emanates from insects’ net effects, making it impossible to rule out the functional importance of edible insects in decomposition (83).

### 7.1. Reduction of food waste

The loss and wastage of food threaten the sustainability of our food system. Millions of tonnes of food waste are generated annually, with research confirming up to 50% waste along the food supply chain (84). Insect-based bioconversions through a novel approach could be an immediate approach to reducing food waste (85). Edible insects can convert low-value food waste such as brewery grains, potato peels, and expired food into biomass and frass for other purposes (84). Using food waste to rear insects is an alternative means to close the gap in the food value chain. Edible insects reared on food waste enter the food chain, with their residues serving as a nutrient source for crop production (86).

### 7.2. Food chain/web

Insects are considered rich in essential nutrients and have recently attracted attention as food and feed for terrestrial livestock or fish (87). Edible insects like *H. illucens* can transform lost nutrients into the food chain as protein-rich human food, animal feed, and even fertilizer (88). Although these edible insects render such tremendous ecosystem services, the fact that they are part of the food chain cannot be ignored. Therefore, they can be harvested and consumed cautiously without overexploiting them beyond their regeneration capacity.

## 8. Economic benefits

Even though edible insects are for human consumption, it is critical to note that the food industry has historically been a significant driver of economic growth and employment creation, making insect farming a promising strategy for alleviating poverty. Insects can be a source of income for even the lowest sections of society because they are easy to harvest, cultivate, rear, and process (89–92). The market for edible insects is expected to expand from $400 million to 1.2 billion in 5 years (2018–2023) (93). More specifically, in the Asia Pacific, the market is likely to exceed $270 million by 2024 (94).

In Africa, lepidoptera is the most consumed order of insects (95). They provide proteins, fats, and essential micronutrients. In South Africa, Uganda, and Nigeria, rearing and selling caterpillars generate income in rural areas (95). In East Africa, insect rearing is rapidly growing and becoming a sustainable option as opposed to the current farming options, which demand arable water and land (96). The authors further indicated the ongoing trends in insect farming, the important insect species, the nutrients derived from insects, and the marketing and regulatory frameworks associated (96). They highlighted how insect farming had created microenterprises in East African countries, including Kenya, Uganda, and Tanzania (96). Insect rearing can reduce poverty as it demands labor. In Africa, after palms have been cut down in a labor-intensive process to extract sap, the cut trunks are revisited to extract the larvae of the palm weevil, *R. phoenicis* (7).

As a result of insect farming practices, new employment opportunities have emerged. Producing powders from insects can generate jobs and financial gain (97). The European Union has recently permitted the use of processed insects as feed for fish (97). Several species of insects can be farmed: *Musca domestica* (L.), *Alphitobius diaperinus* (Panzer), *H. illucens*, *Gryllodes sigillatus* (Walker), *Gryllus assimilisit* (Fabr.), *T. molitor* (L.), *A. domesticus* (L.) (97). Due to rising food prices, the European Union plans to encourage insect rearing and expand the number of insects that can legally be added to fish food. Some African countries, including Uganda and Kenya, feed their poultry with insects (97). If other African countries followed suit, the outcome for the continent would be sustainable development within the animal farming sector.

A recent study showed how insects create socioeconomic changes, mitigate societal challenges, create healthier food, and reduce animal waste production and consumption (98). In Thailand, insect rearing has been revolutionized by disseminating knowledge and improving rearing methods as opposed to the previous practice of collecting insects in the wild (98). Globally, insect rearing has the potential to help humanity achieve the seventeen Sustainable Development Goals (SDGs) proposed by the United Nations. Some of the goals, include zero hunger, good health and well-being. The increasing popularity of eating insects suggests that they could be further promoted as a healthy and sustainable food option.

## 9. Edible insects as pests of crops

Some insect pests are simultaneously considered a vital source of micro-nutrient and protein and thus are consumed by humans. The most common insect pests considered edible include species belonging to the order Coleoptera, Lepidoptera, Hemiptera, and Orthoptera (Table 3) (99, 102). The most well-known edible insect pests are *Schistocerca gregaria* (Forsskål), *L. migratoria*, *Locusta napardalina* (Walker), *Zonocerus variegatus* (L.) and *Nomadacris septemfasciata* (Audinet-Serville) (102). These insect pests can cause significant yield losses to host crops exposing bare ground to soil erosion and impacting ~10% of humans (102). In addition, yam, banana, cassava, cocoa, citrus, cowpea, maize, and soybeans are all targets of these polyphagous feeders. Because of their high nutritional value, locusts are collected and used as food and feed in 65 countries, primarily in Africa and Asia, during outbreaks (53). *Oryctes rhinoceros*, *O. monoceros*, *O. boas*, and *R. phoenicis* are considered an economically important pest of *Elaei guineensis*, *Phoenix dactylifera*, *Raphia* spp. and *Cocos nucifera*. The larvae of *Orycte boas* destroy the crops and cause low yields. Moreover, *R. phoenicis* are voracious feeders, and with their hard mouth parts, they penetrate and damage the plant tissues, causing the leaves to die (106). The larvae of these pests are considered edible insects mainly in Africa (107). The fall army worm *Spodoptera frugiperda* (Smith) and *S. exempta* can destroy entire crops by feeding on the early stages of the maize plant. The larvae of the pest are consumed in Zambia (102). Although insect pests are considered a significant constraint of crop production, edible insect pests have an exceptionally high potential to contribute to a more sustainable and socially equitable global food security. Also, consuming these insects could be an environmentally friendly strategy for biological control.

**Table 3:** Common insect pests used as food.

Insect species	Common name	Consumption stage	Host plant	Reference
Coleoptera				
*Heteroligus mele* (Billberg)	Yam beetles	Adult	*Dioscorea* spp.	(99)
*Rhynchophorus phoenicis* Fabr.) *Oryctes monoceros* (Oliv.)	African palm weevil Coconut beetle	Larvae	*Elaeis guineensis* *Phoenix dactylifera* *Raphia* spp., and *Cocos nucifera*	(100)
Lepidoptera				
*Leuconodes laisalis* Walker	Egg fruit borer	Larvae	*Solanum anguivi, Solanum incanum, Solanum linnaeanum, Solanum macrocarpon, Solanum melongenum, Lycopersicon esculentum and Capsicum annuum*	(101)
*Spodoptera frugiperda* (Smith) *Spodoptera littoralis* (Boisduval)	The fall armyworm African cotton leafworm	Larvae Larvae	*Zea mays and* *Ricinus communis*	(102, 103)
*Cirina forda*		Larvae	*Vitellaria paradoxa*	(102)
Hemiptera				
*Agonoscelis versicolor* (Fabr.)	Sudan millet bug	Adult	*Sorghum* spp.	(104)
Orthoptera				
*Ruspolia* spp. *R. differens* (Serville) *R. nitidulis vicinus* (Walker)	Long-horned grasshopper	Adult	*Ageratum conyzoides* (L.), *Citrus depressa Hayata, Cynodon dactylon* (L.), *D. gayana, Eragrostis mexicana Hornem, Eucalyptus saligna SM., Indigofera arrecta Hochst. ex A. Rich., Persicaria nepalensis* (L.), *and Sorghum halepense* (L.)	(13)
*Kraussaria angulifera* (Krauss)	Senegalese grasshopper	Nymph and adult	*Cenchrus americanus*	(105)
*Nomadacris septemfasciata* (Audinet-Serville)	Red locust	Adult	*Echinochloa pyramidalis, Cynodon dactylon and Cyperus* spp.	(13)
*Schistocerca gregaria* (Forsskål)	Desert locust	Adult	Sorghum spp., *Vigna unguiculata,* *Manihot esculenta,* *Abelmoschus esculentus and Zea mays*	(28)
*Acrida turrita* (L.)	Long-headed grasshopper	Adult	*Vigna unguiculata,* *Manihot esculenta and* *Abelmoschus esculentus*	(28)
*Zonocerus variegatus* (L.)	Variegated grasshopper	Adult	*Chromolaena odorata,* *Vigna unguiculata,* *Manihot esculenta and* *Abelmoschus esculentus*	(102)

## 10. Edible insects as a reservoir of diseases

Insects as food and feed have recently attracted tremendous attention due to their high-quality nutrient contents, ability to upcycle low-grade organic substrates into high-quality insect biomass, and reduction in environmental footprint (94). Despite the rapidly expanding insect farming industry, there has been a significant focus on the potential for disease outbreaks in insect colonies and their spread to humans, animals, and plants.

Insect-borne pathogens threaten the health of humans, animals, and insects as they can cause disease or even death and eventually collapse an entire insect colony (108). A recent study characterized bacterial communities associated with *A. domesticus* and *G. assimilis* and compared populations associated with the surface and whole body of crickets to uncover potentially beneficial and pathogenic microorganisms. Findings from the study support the use of probiotics composed of microorganisms already present in the human digestive system (109). In contrast, some potentially dangerous microorganisms were in the samples.

A recent study by Gałęcki and Sokół (110) identified parasites colonizing mealworms, house crickets, cockroaches, and migrating locusts in Central Europe household farms and pet stores (Table 4). The study revealed parasites in 244 out of 300 examined insect farms. Interestingly, 206 of the cases had parasites that were pathogenic for insects only; 106 had parasites pathogenic for animals; and in 91 cases, parasites were pathogenic for humans (6). However, in humans, before being consumed, edible insects must first undergo one of four common processes: boiling, drying, toasting, or frying (103), which can kill pathogens associated with the edible insect.

**Table 4:** List of diseases/pathogens transmitted by edible insects.

Vector	Pathogen group	Type of pathogen	Pathology notes	Reference
Crickets	Bacteria	Acinetobacter, Enterococcus	Water- or food-borne disease in humans	(109)
Crickets	Bacteria	*Bacillus cereus*	listed as biologically hazardous in edible insects	(111)
Crickets and locust		Gordiidae	Intestinal parasites	(114)
Crickets and locusts	Fungi	*Nosema* spp.	Decrease dry matter consumption, increase mortality in mass-reared insect colonies and reduce profitability	
Cockroaches	Protozoans	*Gregarine* spp.; *Nyctotherus* spp.	Deprive insects of nutrients and compromise the immune system, reproduction and lifespan	
Cockroaches	Nematode	*Thelastoma* spp.; *Hammerschmidtiella diesigni* (Hammerschmidt)	Lower fat content of insect body	
Crickets and locusts	–	*Steinernema* spp.	Have a special structure for storing the bacteria. Once inside the insect’s body, the bacteria are released that produce toxins, which kill the insect.	
Mealworms, house crickets, cockroaches and locusts	Protozoans	*Cryptosporidium* spp.	Cause chronic diarrhoea in reptiles	
Mealworms, house crickets, cockroaches and locusts	Protozoans	*Isospora* spp.	Cause isosporiasis in both immunosuppressed humans and in animals who ingest oocytes	
Mealworms, cockroaches and locusts	Protozoans (Ciliates)	*Balantidium* spp.	Can cause balantidiasis in humans and animals	(17)
Mealworms, cockroaches and locusts	Amoeboids	*Entamoeba* spp. (*E. histolytica* and *E. invadens*)	Can cause dysentery in humans, animals, reptiles, and amphibians	(17)
Mealworms, house crickets, cockroaches and locusts	–	Cestoda	Colonize insects and aids in transmitting tapeworms to birds, insectivorous animals, and humans	(110)
Mealworms and cockroaches	Nematodes	*Pharyngodon* spp.	Colonize wild and captive animals, e.g., lizards	
Mealworms, house crickets, cockroaches and locusts	Nematodes	*Physaloptera* spp.	Influence insect behavior	
Mealworms and cockroaches	–	Spiruroidea	Colonize mainly animals, but can also infec thumans who consume infected intermediate hosts	
Mealworms and cockroaches	Thorny-headed worms	Acanthocephala	Decreases immune reactivity in cockroaches. Can compromise glycogen and lipid levels in crustaceans	
Cockroaches	Arthropods	Pentastomida	Causes pentastomiasis in wild and captive reptiles	
Black soldier fly larvae/rearing substrates	Bacteria	*Staphylococcus aureus*, *Clostridium*, *Escherichia coli, Salmonella and* *Enterococcus*	Could be pathogenic to livestock fed contaminated feed supplements (larval meal)	(112)
Frass of black soldier fly larvae	Phytopathogenic fungi	*Cercospora*	Cause irregular and unhealthy leaf appearance in lettuce plants	(113)

Often, organic side streams are used in rearing Black Soldier Fly (BSF) larvae which are a potential source of food-safety-related microbes (112, 115, 116). In plants, Dzepe et al. (113) identified phytopathogenic fungi (see Table 4) in the leaves of lettuce *Lactuca sativa* when grown in frass-exposed soil. The presence of pathogens in a rearing substrate could be problematic when the resulting residues are used as fertilizers since phytopathogenic fungi can negatively impact crop yield (117). Thus, pathogen contamination of rearing substrates could pose multiple risks when the insects are reared for food and feed and the residue for soil enrichment. Interestingly, BSF larvae can reduce pathogen abundance in substrates during rearing. This pathogen inhibitory effect has been observed for different food-safety-related bacteria, including *Escherichia coli* and *Salmonella* species (115, 118). Kuznetsova et al. (116) found a complete elimination of mycelial fungi from feed substrates when BSF larvae were reared on food substrates.

So far, adult BSF has not been reported as a disease vector. However, van Huis (7) warns against a possible susceptibility to infections following the sector’s rapid growth. Adult House fly (HF) is a nuisance to humans, animals, and vectors of about a hundred pathogens, including bacteria, protozoans, helminths, and viruses (7). Although insect-borne pathogenic viruses are largely host-specific (119), their effects can be felt in humans and other animals that are exposed to disease-ridden material. For instance, adult HF can potentially act as a vector of the orf virus *Ecthyma contagiosum* (Poxviridae), which causes ecthyma in sheep and goats and humans exposed to disease-infected animals (120). Like BSF, HF larvae can reduce the microbial load in manure (14). Furthermore, bacterial endospores have been identified in yellow mealworms and house crickets. A high endospore count of 5.0 log (c.f.u. g − 1) was recorded in cricket samples (121). The study hinted at a possible food safety risk since this endospore count surpassed the lower threshold for *Bacillus cereus* in edible insects. However, no legal microbiological criteria existed specifically for edible insects (121). Therefore, edible insects can sufficiently contribute to diversifying and securing global food and feed. However, the potential of this mini-livestock to harbor and transmit diseases cannot be neglected as they can pose both direct and indirect health risks to humans, animals, and plants.

## 11. Environmental impacts of edible insect production

Safe food and water supply, unpolluted air, safe use of chemicals, sound agricultural practices, and preservation of natural resources are all attributes of a healthy environment and align very well with the United Nations Sustainable Development Goals (SDGs), especially SDGs 6, 12–15 (122). Providing food for everyone now and more so in the future is one of the most significant challenges directly involving SDGs 1 and 2 (98). Our existing food systems are heavily involved in many environmental issues, such as greenhouse gas emissions, eutrophication of freshwater resources, and biodiversity loss (123).

Farming insects for food and feed has recently received considerable attention as a sustainable alternative to conventional food production models, providing food for humans and animals with minimal environmental footprint (Table 5) (97). To understand the comparative advantage of insect farming over conventional livestock farming, Skrivervik (124) re-echoed the criticism meted out on meat production due to its negative impact on the ecosystem. For instance, farming livestock takes up considerable agricultural land, and the emission of nitrous oxide is concerning, thus making livestock production highly eco-degrading (124). Insects have a smaller feed conversion ratio than cattle and require less space to farm. House crickets are known to be about four times more efficient feed converters than pigs and over 12 times better than cattle (124). Almost (in most cases 100%) all of the insect body is consumed, compared to lower than 50% for cattle, which may translate to less food wastage in favor of insects (124). Furthermore, producing insects in areas with near-optimal environmental conditions, such as the tropics, could benefit energy use reduction. Insects are poikilothermic and adjust their body temperature to that of their surroundings, hence less demand for external energy inputs.

**Table 5:** Advantages of insect farming over livestock farming.

Criteria	Insect farming	Livestock farming
Land use	Low requirements for land	High requirements for land
Water use	Low requirements for water	High requirements for water
Pollutants	Low production of greenhouse gases	Greenhouse gases such as carbon dioxide and methane are emitted
Recycling	Transforms low-value matter into nutritious insects by feeding	Some livestock such as cattle do not feed on low-value matter
Use as feed	Insects can be used as fishmeal	Most livestock cannot be used as fishmeal

Madau et al. (97).

Among several factors considered in assessing environmental impact is methane release, which results from the fermentation of Methano-bacteriaceae in the gut, food conversion, and reproduction rate, all typical in beef cattle, poultry, and pigs. This far, cockroaches, termites, and beetles are known to release methane, and less so for other edible insects. For instance, the yellow mealworm is not known to produce methane and thus retains a low global warming potential relative to other livestock products (125). Greenhouse gas emissions from mealworms, house crickets, black soldier flies, and houseflies; feed conversion efficiency, organic waste reduction, and; fishmeal replacement by insect meal in animal feed are all topics that have been examined in the context of conventional livestock production (61).

Despite the several benefits of insect farming, the fast-growing and innovative sector is not without negative effects. Quang Tran et al. (126) suggests a trade-off in using insect meal from BSF, HF, mealworm, and (*Z. variegatus*) as an aquafeed. In their analysis, Quang Tran et al. (126) showed that the inclusion of insect meals in aquafeeds led to higher values of global warming potential, and water and energy use than those obtained in diets without insects. They, however, attribute this impact to the insufficiency of production technology and scalability. Nikkhah et al. (62) also found positive impacts of farming *Protaetia, brevitarsis seulensis* larvae, noting beneficial environmental effects on land use, mineral extraction, and aquatic and terrestrial ecotoxicity when insects were reared on bio-waste. However, several reports found negative environmental effects of insect farming associated with global warming (62, 125).

Edible insect farming can contribute to a sustainable food and feed system, given that some insect species can thrive on low-grade organic streams. However, the safety of derived food and feed cannot be guaranteed without careful monitoring and implementation of preventive measures while closely checking the effects of technological advancements (61).

Edible insect farming has the potential to benefit humanity and the environment; however, farming should be critically reviewed so as not to damage the environment. Farmed insects are numerous, and the order with the highest number of edible insects belongs to Coleoptera (127). There are currently twelve living orders of aquatic insects, six of which are considered edible. Unfortunately, these insects are not being harvested sustainably and are exposed to overexploitation and extinction (127). Dragonflies, for example, are edible aquatic insects, and their over-exploitation and extinction can affect the environment due to an ecosystem balance. It is important to emphasize how farming insects help improve conservation directly and indirectly. Dragonflies feed on mosquitoes, and the former is considered the natural enemy of the latter. The balance between these natural enemies and the pests enhances the environmental health and sustainability of ecosystems and guarantees that there is food at the different trophic levels of the food chain.

The United Nations has reported the urgency of reducing greenhouse gases to combat climate change and improve environmental health (98). Livestock production accounts for about 14.5% of all greenhouse gas emissions (128). Insects raised from human waste can help clean waterways, one of many ways to improve environmental health (98). Several greenhouse gases are currently contributing to the damage of ozone layer. Even though some insects, such as cockroaches, termites, and beetles, release methane into the atmosphere, insect rearing can reduce climate change as it is more environmentally friendly source of protein (98). Even though pig, poultry, and beef products are currently the preferred source of animal proteins, insect rearing has a better impact on the environment regarding reproductive rate, food conversion efficiency, and methane production. The yellow mealworm, *T. molitor*, is considered environmentally benign due to its high reproductive rate and lack of methane production (125).

Many studies have shown that raising insects positively affects the planet (97). Their requirements for arable land and water are relatively low compared to fish or poultry farming (97). In addition, insect rearing has a low environmental cost for producing greenhouse gases such as carbon dioxide (97). Furthermore, insects’ nutritional quality is relatively high compared to the trade-off regarding the effect on the environment. Proteins and minerals like calcium, iron, and zinc are a few nutrients that can be obtained from eating insects. Finally, just as beekeeping has been promoted over several centuries to increase honey production and environmental conservation (129), there is a need for sustainable rearing of edible insects.

## 12. Implication of harvesting wild edible insects on their conservation

Over-exploitation of insects significantly contributes to the decline of many edible insect species, threatening wild insects (73). Usually, insects are sourced through wild harvesting, farming, and semi-domestication of the wild species. However, literature shows that about 92% of these insect species are harvested from the wild (28). Wild harvest of edible insects by humans brings about direct competition with other predators, eventually undermining their population viability. Quite many edible insect species are hosts or prey to other organisms. Hence, overexploitation of edible insects from the different trophic levels beyond regeneration capacity may adversely affect the population of other organisms and, consequently, the provision of some essential ecosystem services (130). Over a decade of research has revealed that the population of widely consumed Mopane worm (*Imbrasia belina*) keeps declining in South Africa and Zimbabwe due to increased commercialization and overexploitation (131). The global trade in insect species continues to grow due to the need to feed a world population approaching 8 billion people (132). During periods of meat protein shortage, insects constitute nearly a third of their protein intake, which threatens the edible insect species population since it often exceeds their regeneration capacity. Also, the collection practices have become less selective and sustainable (133). A decline in the population of some edible insect predators or parasitoids has been reported in western and northern Europe and New Zealand (134).

## 13. Conclusion and future perspectives

Using insects as food and feed has a long history since ancient times and continues to provide food for millions of people worldwide. Edible insects contain essential nutrients, such as carbohydrates, proteins, vitamins, and minerals, which have antimicrobial properties. Apart from these benefits, edible insects require a smaller space for production than livestock, which need more extensive land to produce the same amount of energy. Edible insects provide many ecosystem services, such as decomposition, pollination, reduction of food waste and support of food chain or web, and monitoring of environmental health. Utilizing insects as food and feed, we can alter beef, fish, and poultry consumption and the life cycle assessment, thereby reducing greenhouse gas emissions, ammonia emissions, and carbon footprints. Several activities associated with edible insect production and marking generate jobs, and income, thereby ensuring poverty reduction and zero hunger, especially in developing countries. Edible insects can potentially improve our global food security significantly, but they also have several challenges that need addressing. Some wild edible insects harbour pathogens of plants, animals, and humans. For instance, migratory locusts are consumed by amphibians, reptiles, and humans, mainly in parts of Africa and Asia. *Nosema* spp. and *Gregarine* spp., which cause severe losses to bee colonies worldwide, are the common parasites of Locusts. *Acheta domesticus* harbors *Nosema* spp., *Gregarine* spp., and *Steinernema* spp. (135). Though *A. domesticus* are often consumed in powdery form or protein extracts, the insect can also be consumed directly (136, 137). Recent scientific research demonstrates that the bacteria levels and anti-nutrient components in edible insects are reduced using preservation procedures, primarily thermal treatments, employed in cooking or processing (135). Specifically, these methods suggest proper preparation by boiling, drying, toasting, or frying edible insects to ensure a safe diet. Rearing edible insect pests of horticultural and forest crops like termites, locusts, and grasshoppers requires an appropriate procedure to avoid possible introduction outside the farming facility. Moreover, with proper rules and policies, these alternative protein sources may offer a solution to problems of availability and accessibility of conventional proteins sources. Furthermore, the problem of overexploitation of edible insect resources can be curbed by laying rules to control their consumption and, more importantly, by educating people on the need to move from wild harvesting to farming and semi-domesticating wild species. In farming/rearing edible insects, there would be a need to encourage the use of food leftovers to save production costs and solve the problem of loss and wastage of food.

## Author contributions

OFA: Study – conceived and designed, writing – original draft, and review and editing. JO-O, KA, AKD, BOB, SKD, KDN, and SYC: writing – original draft, review and editing. SAS: review and editing. All authors contributed to proofreading of the final version.

## Conflict of interest

The authors declare that the research was conducted in the absence of any commercial or financial relationships that could be construed as a potential conflict of interest.

## Publisher’s note

All claims expressed in this article are solely those of the authors and do not necessarily represent those of their affiliated organizations, or those of the publisher, the editors and the reviewers. Any product that may be evaluated in this article, or claim that may be made by its manufacturer, is not guaranteed or endorsed by the publisher.

## Footnotes

^1^https://www.semanticscholar.org/

^2^https://scholar.google.com

^3^www.scopus.com

^4^https://www.sciencedirect.com/science

^5^https://scielo.org/es/
